# What types of information do pharmacists include in comprehensive medication management review reports? A qualitative content analysis

**DOI:** 10.1007/s11096-023-01561-5

**Published:** 2023-03-17

**Authors:** Tarik Al-Diery, Hollie Freeman, Amy Theresa Page, Amanda J Cross, Deborah Hawthorne, Kenneth Lee

**Affiliations:** 1 College of Pharmacy, Qatar University, Doha, Qatar; 2 Centre for Optimisation of Medicines, Discipline of Pharmacy, School of Allied Health, University of Western Australia, Perth, Australia; 3 Western Australian Centre for Health and Ageing, School of Allied Health, University of Western Australia, Perth, Australia; 4 Consultant Pharmacist Services Research Network, Coherent, Australia; 5 Centre for Medicine Use and Safety, Faculty of Pharmacy and Pharmaceutical Science, Monash University, Parkville, Australia

**Keywords:** Content analysis, Medication review, Medication therapy management, Pharmacy practice, Qualitative research

## Abstract

**Background:**

Comprehensive medication management reviews are an established intervention to identify medication-related problems, such as the prescribing of potentially inappropriate medications, and under- and over-prescribing. However, the types of information included in written reports of comprehensive medication management reviews, beyond types of medication-related problems, are unknown.

**Aim:**

This study aimed to explore the types of information Australian pharmacists include in their written reports following comprehensive medication management reviews.

**Method:**

Australian consultant pharmacists were invited to upload their 10 most recent written reports of their domiciliary-based comprehensive medication management reviews. A random selection of the reports, stratified by each pharmacist, were included for qualitative content analysis.

**Results:**

Seventy-two de-identified reports from eight consultant pharmacists located in five of the eight Australian States and Territories were included for analysis. From the evaluated reports, four major categories of information were identified: (1) patient details such as date of interview (n = 72, 100%) and medicine history (n = 70, 97%); (2) pharmacist assessment including assessment of the patient (n = 70, 97%), medicines management (n = 68, 94%) and medicine-related issues (n = 60, 83%); (3) pharmacist recommendations, specifically pharmacological recommendations (n = 67, 93%); and (4) patient-centred experiences such as perspectives on medicines (n = 56, 78%). Reporting of patient-centred experiences appeared most variably in the included reports, including patient concerns (n = 38, 53%), willingness for change (n = 27, 38%), patient preferences (n = 13, 18%), and patient goals (n = 7, 10%).

**Conclusion:**

Pharmacists within our study included a wide variety of information in their comprehensive medication management review reports. Aside from medication-related problems, pharmacists commonly provided a holistic assessment of the patients they care for. However, variability across reports has the potential to impact consistent service delivery.

## Impact statements

- The holistic nature of the medication management reports suggests a broader role for pharmacists beyond medication-related tasks and could encompass championing the patient perspective.
- Variability in the reporting of medication management reports could impact consistent service delivery, and thus variability in patient outcomes.

## Introduction

The high prevalence of polypharmacy and medication-related problems in older patients with multiple comorbidities places importance on optimising interventions to improve medication use [1–4]. Comprehensive medication management reviews are a clinical service offered by pharmacists with the aim of identifying and resolving potential medication-related problems and optimising evidence-based therapeutic care [5–9]. Medication reviews have been found to reduce medication-related problems such as drug-drug interactions and optimisation of pharmacotherapy, but the extent of the effectiveness on clinical outcomes, hospital admission, and mortality remains unclear [3, 10–18]. Nonetheless, globally, pharmacist-led medication reviews have been effective in promoting patient-centred care through an interprofessional approach[19].

While medication review processes vary internationally on how information is collected and presented, the focus of medication reviews is generally intended to be patient-centred in addressing key medical and social issues [6–8]. The Australian ‘Home Medicines Review’ (HMR) service is designed to target the management of high-risk patients who may have multiple comorbidities, issues with medication non-adherence, or are not optimised in their care from an evidence-based medicine approach [20, 21]. In Australia, HMRs are a government-funded collaborative service that is undertaken by a consultant pharmacist upon referral from a general practitioner (GP) [20, 22]. In Australia, consultant pharmacists are registered pharmacists who have undergone additional approved training in performing comprehensive medication management reviews [20]. Consultant pharmacists undertake a multifaceted process that often involves a prior evaluation of medical and social history, an interview in the home, followed by an written report with recommendations to the GP[20].

Despite medication management reviews being extensively utilised in Australia and in other parts of the world for over 20 years, current literature on medication management reviews mainly focuses on the impact a pharmacist has on direct or surrogate health outcomes with varying results [17, 21]. Surrogate outcomes include identifying medication-related problems and the recommendations made as a result, and the perceptions of patients and GPs toward the medication review process [17, 21, 23–33]. Other retrospective studies have evaluated the types of medication-related problems identified, the prevalence of their identification, and the subsequent acceptance and implementation of medicines review pharmacist recommendations by the GPs [10, 15, 17, 28].

While previous studies have reported on the impact that pharmacists have on health outcomes through some of the content they include in their medication review reports, there is a gap in understanding the types of information included by pharmacists in medication management review reports, beyond medication-related problems, and the frequency of reporting [17, 21].

### Aim

The present study aimed to explore the types of information Australian pharmacists include in their written reports following comprehensive medication management reviews.

### Ethics approval

This research was approved by the Human Ethics Office of the University of Western Australia, approval number 2021/ET000392.

## Method

### Study design

The study sought to analyse retrospectively written HMR reports by consultant pharmacists for patients living in the community. Participants were invited to de-identify and upload their 10 most recent HMR reports and provide answers to demographic questions via an anonymous online questionnaire. Each report was screened by one author (KL) to ensure correct de-identification procedures. As not all participants uploaded 10 reports each, a random selection of the reports, evenly stratified by each participant (to ensure equal number of reports selected for analysis), were included for qualitative content analysis. A content analysis approach was chosen to allow for broad exploration of the types of information included in a medication management review report, as well as quantification of qualitative data to facilitate characterisation of information types.

Participation was voluntary and no reimbursements were able to be offered despite requesting participants to dedicate time to finding, de-identifying, and uploading their reports. Given the time requirements, the authors did not specify an a priori target sample size, nor aim for data saturation. Rather, the authors sought to gain insight into the range of information types included in medication management review reports. Multiple reports from each participant were sought to capture potential for variability within individuals.

### Participants and settings

Participants were eligible if they were an Australian consultant pharmacists credentialled with one of the accrediting organisations and had completed at least one HMR within the past 12 months at the time of the study. At the point of data collection, the Australian Association of Consultant Pharmacists (AACP) and The Society of Hospital Pharmacists of Australia (SHPA) were the two organisations responsible for the accreditation of pharmacists to undertake medicine management reviews [34]. To gain accreditation, pharmacists must be currently registered pharmacists, show evidence of fulfilling specified continued professional development, and have completed a competency-based assessment relevant for medicine management reviews [34]. At the time of writing, there are over 2000 accredited pharmacists across all Australian States and Territories [35, 36]

### Recruitment

Participants were recruited over a 10-week recruitment period from June to August 2021. To capture the practices of pharmacists across Australia, participants were recruited using an online advertisement, which was promoted through several means including social media and email correspondence. The advertisement was shared in private Facebook groups, comprising of Australian consultant pharmacists, as well as on the professional LinkedIn and Twitter profiles of the research team. It was also sent to accrediting bodies (AACP and SHPA), medicine review support services, and personal contacts of the research team via email. Participants were eligible for the study if they were consultant pharmacist practising in Australia. Participants who were either not Australian consultant pharmacists or had not completed at least one HMR service in the past 12 months were excluded from the study.

### Data analysis and quality assurance

The de-identified reports were imported into NVivo (version 12 Plus) and coded inductively [37, 38]. To create an initial analytical framework, one report was selected at random, independently coded by two authors (HF and KL), and then compared. Discrepancies were resolved through team discussion and the analytical framework was updated accordingly. One author (HF) then coded all remaining reports independently using the initial analytical framework. Any emerging codes beyond the initial analytical framework were added to the framework by HF, following discussion with KL, an experienced qualitative researcher and consultant pharmacist. The finalised analytical framework was then independently applied to 10% of the total reports by KL. Discrepancies were compared and resolved through team discussion.

Quality assurance of the analytical process was established in a number of ways [39]:

(A) Credibility was established through analyst triangulation between HF and KL, and peer debriefing with an audience of pharmacy academics and students.
(B) Dependability and confirmability were established through the development of an audit trail to record all coding decisions, as well as via analyst triangulation.

## Results

Twenty-four pharmacists responded to the advertisement of interest for inclusion into the study, of which nine pharmacists uploaded de-identified reports and completed the demographic survey. The written documents uploaded by one pharmacist were excluded as they were for people residing in residential aged care facilities. Two pharmacists uploaded only nine reports, giving a total of 88 submitted de-identified reports. After stratified random selection, nine reports from eight pharmacists (a total of 72 included reports) were included for analysis. Most participants were experienced pharmacists with greater than ten years of work experience and working in the metropolitan area. A summary of the pharmacist demographics can be seen in Table 1.

**Table 1:** Participant demographics (n = 8)

Participants’ demographics	Options to select	n (%)
Age	61 years +	3 (38)
	51–60 years	3 (38)
	31–40 years	2 (25)
Gender	Female	5 (63)
	Male	3 (38)
State	Victoria	4 (50)
	Queensland	1 (13)
	Western Australia	1 (13)
	New South Wales	1 (13)
	Tasmania	1 (13)
Geographical setting	Metropolitan	6 (75)
	Rural	1 (13)
	Metropolitan + rural	1 (13)
Highest level of education achieved	Bachelor’s degree	4 (50)
	Graduate certificate	1 (13)
	Master’s degree	1 (13)
	Doctorate degree	2 (25)
Accreditation achieved through	Australian Association of Consultant Pharmacists only	6 (75)
	Society of Hospital Pharmacists of Australia only	0 (0)
	Australian Association of Consultant Pharmacists AND Society of Hospital Pharmacists of Australia	2 (25)
Years as a registered pharmacist	No response	1 (13)
	0–9	1 (13)
	10–19	1 (13)
	20–29	0 (0)
	30–39	2 (25)
	40–49	1 (13)
	50–59	2 (25)
Years accredited as consultant pharmacist	0–9	4 (50)
	10–19	2 (25)
	20–29	2 (25)
Average number of Home Medicine Reviews conducted a month	0–5	4 (50)
	6–15	2 (25)
	16–20	2 (25)

**Fig. 1:**
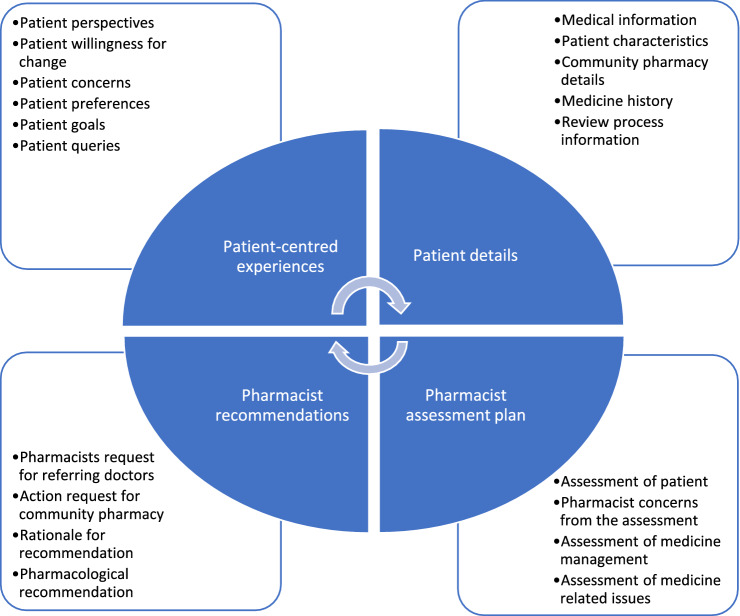
Major categories and corresponding codes of the written report content analysis

We identified four major categories of information included in the HMR reports: patient details, pharmacist assessment plan, pharmacist recommendations, and patient-centred experiences. Figure 1 displays the four major categories and respective codes.

### Patient details

Participants recorded multiple details such as the medical, medicine, and social history, along with other factors such as patient characteristics and review process details (see Table 2). The following types of information were commonly found across the reports: current medicine history (97%), medicine administration (82%), medical history (81%), clinical parameters (78%), any other healthcare practitioners involved (67%), and allergies and adverse drug reactions (ADRs) (57%) in at least one of their reports. The following types of information were less commonly found across the reports: documentation of diet and exercise (49%), smoking status (38%), and alcohol status (36%).

**Table 2:** Patient details from written reports (n = 72)

Code and subcodes	Number of written reports containing each type of information n (%)
1. Medical information	58 (81)
Clinical parameters	56 (78)
Laboratory and pathology data	44 (61)
Tests and observations	34 (47)
Medical history	58 (81)
Medical conditions	47 (65)
Vaccination history	22 (31)
Family medical history	5 (7)
Other involved health care practitioners	48 (67)
Allergies and adverse drug reactions	41 (57)
Biometric information	20 (28)
Diet and exercise	35 (49)
Smoking status	27 (38)
Alcohol status	26 (36)
Details of family and living situation	22 (31)
Employment	6 (8)
Hobbies	3 (4)
2. Patient characteristics	2 (3)
3. Community pharmacy details	48 (67)
4. Medicine history	70 (97)
Changes to medicines	53 (74)
Medicine administration	59 (82)
Medicines taken currently	70 (97)
Medicines taken in the past	34 (47)
Medicines not started yet	5 (7)
5. Review process information	72 (100)
Date of interview	72 (100)
How review was conducted	62 (86)
People present during the interview	16 (22)
Reason for referral	30 (42)
Information from previous medicine review	10 (14)
Date of referral	15 (21)

### Pharmacist assessment plan

Assessment of the patient was the most reported information type (97%), followed by an assessment of the medicines (94%), then assessment of medicine related issues (83%). Table 3 demonstrates that participants assessed the patient in many ways. Of note, the most included information in reports were the participants’ assessment of potential medicine issues (75%), medicine and device knowledge (74%), symptoms (74%), and adherence with taking medicines (74%). Medicine management and storage at home was less commonly included, with 40% of reports assessing this point.

**Table 3:** Pharmacist assessment plan from written reports (n = 72)

Code and subcodes	Number of written reports containing each type of information n (%)
Assessment of patient	70 (97)
Assessment of symptoms	53 (74)
Medicine and device knowledge	53 (74)
Information and treatment received from other health care practitioners	26 (36)
Actions to improve condition	13 (18)
Knowledge of health conditions	7 (10)
Pharmacist concerns from the assessment	13 (18)
Pharmacist concerns about patient	13 (18)
Pharmacist observations of patient during the interview	12 (17)
Assessment of medicine management	68 (94)
Adherence with taking their medicines	53 (74)
Comments on medicine management	47 (65)
Medicine management and storage at home	29 (40)
Assessment of medicine related issues	60 (83)
Potential medicine issue	54 (75)
Actual medicine issue or adverse effect	26 (36)
Suspected medicine issue	18 (25)

### Pharmacist recommendations

As shown in Table 4, pharmacological recommendations were made in 93% of reports. Recommendations to commence or re-commence a medicine (74%) were more common than recommendations to cease a medicine (49%). Justification for recommendations varied, with 64% providing pharmacological and clinical rationale, but only 49% provided details of the resources and references used to support one or more of their recommendations.

**Table 4:** Pharmacist recommendations from written reports (n = 72)

Code and subcodes	Number of written reports containing each type of information n (%)
Pharmacists request for referring doctor	38 (53)
Doctor to update medical records	17 (24)
Provide updated information to other health care practitioners	18 (25)
Request for GP management plan	16 (22)
Action request for community pharmacy	4 (6)
Rationale for recommendation	58 (81)
Pharmacological and clinical rationale	46 (64)
Resources and references used	35 (49)
Supporting information around criteria for subsidised prescribing	4 (6)
Pharmacological recommendation	67 (93)
Change dose of medicine	39 (54)
Consider commencing or recommencing a medicine	53 (74)
Cease medicine	35 (49)
Modify current medicine management	18 (25)
Administration aid recommended	6 (8)

### Patient-centred experiences

Table 5 summarises the frequency of including information pertaining to patient-centred experiences. Of note, the patient perspective was reported in 81% of reports, with the most common sub-type being patients’ opinions about medications (78%). Reporting of other aspects of patient-centred experience was less common, including patient concerns (53%), willingness for change (38%), patient preferences (18%), and patient goals (10%). The least commonly included information sub-type was patients’ perspectives of other healthcare professionals involved in their care (4%).

**Table 5:** Patient-centred experiences from written reports (n = 72)

Code and subcodes		Number of written reports containing each type of information n (%)
Patient perspectives		58 (81)
Perspectives on medicines		56 (78)
Perspectives on health conditions		19 (26)
Perspectives on other treatments received		6 (8)
Perspectives on healthcare professionals		3 (4)
Patient willingness for change		27 (38)
Willingness to change medicine management		18 (25)
Willingness to make lifestyle changes		14 (19)
Patient concerns		38 (53)
Medicine concerns or difficulties		21 (29)
Health concerns or complaints		25 (35)
Patient preferences		13 (18)
Patient goals		7 (10)
Patient queries		6 (8)

## Discussion

The present study adds to extant literature on comprehensive medication management reviews by going beyond characterisation of medication-related problems through exploration of the variety of information types included in medication review reports. We identified in this study that pharmacists reported a wide variety of information, broadly classified into patient details, pharmacist assessment plan, pharmacist recommendations, and patient-centred experiences.

While a recent study [22] evaluated the alignment of HMR reports with best practice guidelines, the inductive approach taken in our study provides further insight into other types of information included in HMR reports, such as a details of family and living situations, medicine management and storage at home, provision of practical prescribing information, and patients’ willingness for behaviour change. Our study has revealed that pharmacists include more information than medication-related information in the HMR reports, and suggests a holistic assessment of patients including social, physical, and behaviour considerations, as evident through the reporting of diet and exercise, and patient concerns, preferences, and goals.

Despite the holistic nature of the reports included in our analysis, the frequency of each information type that was included in the reports varied. Given the inconclusive evidence surrounding the clinical benefits of comprehensive medication management reviews (such as in terms of hospital admissions) [3, 10–18], the variability of reporting identified by our study sheds light into why the evidence of clinical benefit may be inconclusive. This variability was also recently noted by Patounas and colleagues [22]. Furthermore, a lack of a standardised approach in written reporting suggests that there could be inconsistencies in the level of care patients are receiving, as well as the quality of written reports GPs receive. It is possible that one component of the perception and uptake of the medication management review program overall is its consistency and replicability [40, 41]. Standardised question templates for medication management reviews that allow for flexibility in reporting depending on the pharmacist work environment may contribute to a higher consistency in issues found, leading to interventions also having a higher degree of replicability [42]. Nonetheless, obstacles for maintaining consistency in medication management review reports include lack of auditing of the reports and the time-intensive nature of writing medication management reviews which require ongoing pharmacist education and training that may not be readily available in healthcare systems [22].

Participants in our study were consistent in recording patient details and pharmacist assessments such as medical information, medicine history, and assessment of the patient. However, participants were inconsistent in reporting the reasons for the medication management review referral and medicine management and storage at home. The consistency in reporting of this type of information is similar to the findings from an earlier study [22]. Other work has shown that the reasons for the medication management review referral is inconsistent on both the original referral from the GP and the written report [22]. Previous research in the hospital setting of the pharmacist-doctor relationship in clinical reporting suggests that pharmacists traditionally prefer to communicate information verbally rather than through written documentation due to ease in communication and that doctors are not always inclined to read their reports, particularly when they are lengthy [43], and that the service may be perceived as external rather than integrated [41]. Limited GP acknowledgment of written reports has been identified in other settings as a barrier to high quality reports [44]. However, it is improbable that these written reports are supplemented by verbal communication. The reasons for why participants were not consistent in reporting certain information were beyond the scope of this study. As consultant pharmacists have been encouraged to tailor their written reports to the preferences of individual referring GPs, the variation observed may reflect that the experienced consultant pharmacists participating in this study are writing tailored reports.

Medication management review processes in other parts of the world emphasise a patient-centred approach to capture not only existing medicines and medicine related problems, but also the patient experience (social and behavioural concerns), to ensure that full patient medical experience is captured and appropriately addressed [6, 7]. Previous research regarding patient experiences with medication management reviews has largely focused on patient perceptions regarding the benefits and barriers of the medication management review service [33, 45]. Our study demonstrated that a small proportion of reports acknowledge patient concerns, preferences, and goals. However, findings from the DREAMeR-study suggest that clinical medication management reviews that focus on patient goals results in a better quality of life and decreases the number of health problems [46]. The process evaluation the AusTaper study of pharmacist medicines reviews in general practice demonstrated that synergy in facilitating patient understanding and shared decision making was a key component of successful pharmacist reviews [47]. The findings from our study therefore highlights that more work is required to acknowledge patient goals, in order to ensure the full patient-centred experience is being captured.

Future research is needed to understand what factors within the medication management review reports are likely to affect a GPs decision to make an implementation from a pharmacist recommendation, and whether citing references leads to better implementation rates. Ideally, future research would investigate a simulated patient referred by an unknown GP for whom participating consultant pharmacists write a report. The variation in content of the written report could then be assessed independently. Although there is some basic guidance on how to structure medication management reviews, there is minimal literature to inform on the type of information to be included in medication management review reports [17, 20, 28]. A multidisciplinary consensus study between pharmacists and GPs can potentially create a more flexible standardised reporting method that improves consistency in reported information and improve GP implementation rates of pharmacist recommendations.

This study had notable strengths and limitations. Strengths included representation of participants from across five of the eight States and Territories of Australia. Nonetheless, limitations of our research include the small sample size of pharmacists recruited and the number of included written reports. Within the context of a qualitative content analysis, our study reports on valuable patterns of written reports that captures a range of pharmacist behaviours in the medication management review process. While the exact percentages of reported figures will vary from sample to sample, particularly given the small non-random sample size, our study provides first insight into the potential variability. A further limitation of this study is that the reports received were predominantly from pharmacists with many years of experience, both in practice and as consultant pharmacist. Without data from younger, less experienced pharmacists, it is hard to make conclusions reflective of all consultant pharmacists. A study incorporating a larger sample would therefore be recommended to confirm the results found in this study and reduce the biases that have been identified in the current study. Furthermore, while participants were asked to submit 10 of their most recent reports, it is plausible that some participants may select reports perceived as ‘better quality’, thereby biasing our findings. However, several strategies were adopted to mitigate this risk, such as clarifying with participants that the aim of the research is not to judge quality and emphasising the anonymous nature of data collection.

## Conclusion

This study demonstrated that pharmacists include a wide variety of information within their comprehensive medication management review reports, beyond reporting on medication-related problems. Our study suggests that pharmacists perform holistic assessments of patients, taking into consideration social, physical, and behavioural factors; and are practical in their recommendations to support prescribing. Despite the holistic assessment of patients, more work is needed to include patient goals and preferences within the reports. Furthermore, despite the breadth of information included in a comprehensive medication management review report, our study suggests that there is wide variability across reports, which has the potential to impact consistent service delivery.
